# The Mitochondrial Trigger in an Animal Model of Nonalcoholic Fatty Liver Disease

**DOI:** 10.3390/genes12091439

**Published:** 2021-09-18

**Authors:** Guglielmina Chimienti, Antonella Orlando, Francesco Russo, Benedetta D’Attoma, Manuela Aragno, Eleonora Aimaretti, Angela Maria Serena Lezza, Vito Pesce

**Affiliations:** 1 Department of Biosciences, Biotechnologies, and Biopharmaceutics, University of Bari Aldo Moro, Via Orabona 4, 70125 Bari, Italy; guglielminaalessandra.chimienti@uniba.it (G.C.); angelamariaserena.lezza@uniba.it (A.M.S.L.); 2 Laboratory of Nutritional Pathophysiology, National Institute of Gastroenterology “S. de Bellis” Research Hospital, Castellana Grotte, 70013 Bari, Italy; antonella.orlando@irccsdebellis.it (A.O.); francesco.russo@irccsdebellis.it (F.R.); benedetta.dattoma@irccsdebellis.it (B.D.); 3 Department of Clinical and Biological Sciences, University of Turin, Corso Raffaello 30, 10125 Torino, Italy; manuela.aragno@unito.it (M.A.); eleonora.aimaretti@unito.it (E.A.)

**Keywords:** NAFLD, mitochondrial biogenesis, mtDNA damage, DHEA, high fat-fructose diet

## Abstract

Nonalcoholic fatty liver disease (NAFLD) is the leading liver chronic disease featuring hepatic steatosis. Mitochondrial β-oxidation participates in the derangement of lipid metabolism at the basis of NAFLD, and mitochondrial oxidative stress contributes to the onset of the disease. We evaluated the presence and effects of mitochondrial oxidative stress in the liver from rats fed a high-fat plus fructose (HF-F) diet inducing NAFLD. Supplementation with dehydroepiandrosterone (DHEA), a multitarget antioxidant, was tested for efficacy in delaying NAFLD. A marked mitochondrial oxidative stress was originated by all diets, as demonstrated by the decrease in Superoxide Dismutase 2 (SOD2) and Peroxiredoxin III (PrxIII) amounts. All diets induced a decrease in mitochondrial DNA content and an increase in its oxidative damage. The diets negatively affected mitochondrial biogenesis as shown by decreased peroxisome proliferator-activated receptor-γ co-activator-1α (PGC-1α), mitochondrial transcription factor A (TFAM), and the COX-IV subunit from the cytochrome c oxidase complex. The reduced amounts of Beclin-1 and lipidated LC3 II form of the microtubule-associated protein 1 light chain 3 (LC3) unveiled the diet-related autophagy’s decrease. The DHEA supplementation did not prevent the diet-induced changes. These results demonstrate the relevance of mitochondrial oxidative stress and the sequential dysfunction of the organelles in an obesogenic diet animal model of NAFLD.

## 1. Introduction

Nonalcoholic fatty liver disease (NAFLD) is a liver pathology diagnosed in subjects consuming little or no alcohol, not presenting secondary causes of hepatic steatosis [1] and in which at least 5% of hepatocytes are infiltrated with steatosis [2]. NAFLD includes two subtypes featuring different outcomes: the non-progressive form of nonalcoholic fatty liver (NAFL) and the progressive form of nonalcoholic steatohepatitis (NASH). NASH usually presents, besides steatosis, lobular inflammation, and ballooning of hepatocytes with or without perisinusoidal fibrosis [3]. In a small group of patients, it may develop into cirrhosis and hepatocellular carcinoma (HCC) [4], thus requiring individual screening and surveillance [1]. In recent years, NAFLD’s incidence has progressively risen, concurrent with that of diabetes and obesity. NAFLD is nowadays the leading liver chronic disease, affecting one billion persons altogether, and is likely present in roughly 25% of the world’s population [5]. In particular, the disease prevalence doubles in diabetic or obese subjects, and it is no longer limited to Western countries, featuring an unhealthy lifestyle characterized by sedentariness and hypercaloric diet, but has spread to urbanized areas of undeveloped countries [6]. Because of the high incidence of NAFLD in obese subjects, presenting a mild diffuse inflammation that affects insulin signaling and leads to insulin resistance (IR), recently NAFLD has been proposed to constitute the hepatic component of metabolic syndrome, featuring a metabolic derangement of the whole organism [7,8]. Despite the disease’s extensive diffusion, there is no drug-approved therapy, and even the origin of the pathology has not been clearly assessed. The initial “two-hit theory”, according to which accumulation of fatty acids in the liver (the characteristic steatosis) should constitute the first hit, followed by a second one, due to oxidative stress, activating the cascade of inflammatory cytokines that leads to the damage of liver parenchyma [9], has been substituted by the more recent “multiple parallel hits theory” [10]. According to the latter, several “multiple parallel (and not sequential)” hits, including IR, oxidative stress, genetic and epigenetic factors, intestinal microbiota composition, and environmental elements, should cooperate to induce triglyceride accumulation in hepatocytes. This condition results from an imbalance between the presence of fatty acids, due to uptake of diet- or adipocytes-derived fatty acids and “de novo” synthesis, and their elimination, obtained through mitochondrial β-oxidation and secretion by very-low-density lipoproteins (VLDLs) into the blood. Several enzymes and transcription factors are involved in the regulation of lipid metabolism. The steroid regulatory element-binding proteins (SREBPs), fundamental in “de novo” lipogenesis, have been suggested to be relevant in NAFLD origin. In particular, steroid regulatory element-binding protein-1c (SREBP-1c) is crucial in regulating the expression of genes taking part in hepatic triglyceride synthesis and may also be involved in the pathogenesis of NAFLD [11]. Nutritional status and other factors such as insulin levels, liver X-activated receptors (LXR), and Sp1 and nuclear factor Y elements affect the regulation of SREBP-1c [12,13]. It has been demonstrated that saturated fatty acids strongly induce SREBP-1c, likely through their ability to promote inflammation, and that a high-fat diet plus fructose (HF-F), mimicking an unhealthy Western diet, induces SREBP-1c overexpression in rats through diet-induced oxidative stress [14]. Effectively, in recent years, because of the marked relevance of nutrition on onset and progression of NAFLD, several different animal models, in which the applied diet leads to the disease, have been developed. Among such obesogenic models, one based on a high-fat diet plus fructose (HF-F) has previously been analyzed by our group for its involvement of SREBP-1c in the generation of hepatic steatosis and for the induction of liver insulin resistance in rats [14]. In that study mitochondria were not examined, although mitochondria are the cellular base of β-oxidation, which is involved in the derangement of lipid metabolism at the basis of NAFLD. In rodent models of the disease, the increased lipid delivery to the liver following an obesogenic diet raises the activity of TCA cycle and anaplerotic/cataplerotic fluxes, which promote oxidative stress and inflammation [15]. Similarly, increased rates of hepatic mitochondrial respiration are reported in obese human subjects with IR, but this adaptive response is lost in patients who develop NASH. This evidence suggests that alterations in mitochondrial function appear early in the pathogenesis of hepatic IR and steatosis and indicates the need for further analyses of mitochondrial metabolism in the dissection of the mechanisms underlying NAFLD progression [15]. The potentially relevant role played by mitochondria in the pathogenesis of NAFLD is also supported by their involvement in inducing oxidative stress, producing inflammatory cytokines, and in the origin of IR, all cooperating as multiple hits to the disease’s onset. Therefore, in the present study, we decided to evaluate the presence and the effects of oxidative stress in mitochondria from the liver of rats treated with an obesogenic diet inducing NAFLD, already used by us as an animal model, namely, a high-fat plus fructose (HF-F) diet. The effects on mitochondrial DNA (mtDNA) content and integrity, mitochondrial biogenesis, and autophagy were analyzed in the diet-induced NAFLD model and in supplementation with dehydroepiandrosterone (DHEA) added to the diet. DHEA is a compound of physiological origin with multitargeted antioxidant properties that was previously demonstrated to positively affect lipid dysregulation as well as the generation of cell oxidative and inflammatory stress in rats whose HF-F diet was fortified with DHEA [14]. Therefore, DHEA was tested for a potential role in delaying NAFLD through its effects at mitochondrial level thanks to the compound’s generalized antioxidant activity at the applied dosage.

## 2. Materials and Methods

### 2.1. Animals and Experimental Design

Animals were housed at the animal facility of the Department of Experimental Medicine and Oncology, University of Turin, and cared following the Principles of Laboratory Animal Care (NIH No. 85–23, revised 1985) and the Italian Ministry of Health Guidelines (No. 86/609/EEC). The local ethics committee approved the scientific project, including all the applied procedures to minimize animal suffering. Male Wistar rats (Harlan Laboratories, Udine, Italy) weighing 200–220 g, 1-month-old, were divided into five groups, each including four animals: control rats (Ctrl) fed with a standard lab chow (24% protein, 65% carbohydrate, 11% fat), ad libitum water; rats maintained on a standard diet plus 10% (*w*/*v*) fructose dissolved in the drinking water (Fructose); high fat (HF) rats maintained on a 17% protein, 53% carbohydrate and 26% fat plus 4% cholesterol diet; rats fed with the HF diet plus 10% (*w*/*v*) fructose in the drinking water (HF-F); and rats maintained on the HF-F diet fortified with 0.01% (*w*/*w*) DHEA (HF-F-D group). According to the chosen group, animals were fed the selected diet for 15 weeks. Rats were checked every day, and body weight, water and food intake were recorded weekly. At the end of the treatments, the animals were anesthetized with 20 mg/kg (*b*/*w*) of Zoletil 100 (Virbac S.r.l., Carros, France) and sacrificed by aortic exsanguination. Liver samples were immediately removed and stored at −80 °C until assayed.

### 2.2. SOD2 Concentration

The Mitochondrial Superoxide dismutase (SOD2) concentration in liver tissue samples from Ctrl, Fructose, HF, HF-F, and HF-F-D rats was evaluated using the Rat SOD2 Enzyme-Linked Immunosorbent Assay (ELISA) Kit (FineTest, Wuhan, China) following the instructions of the manufacturer.

### 2.3. Western Immunoblotting

Protein extracts were obtained from liver samples of Ctrl, Fructose, HF, HF-F, and HF-F-D rats using standard procedure [16]. Aliquots of 50 μg of total protein from each sample were loaded into 4–15% pre-cast polyacrylamide gels (Bio-Rad, Milan, Italy) for Western blot analysis. Anti-PrxIII (LF-PA0030,Ab FRONTIER, Seoul, Korea), anti-PGC-1α (NBBP1-04676, Ab NOVUS, Centennial, CO, USA), anti-TFAM (74955), anti-COX-IV (48445), anti-Beclin-1 (37385), anti-LC3 (127414), and anti-β-actin (4970) (Cell Signaling, Danvers, MA, USA) were used as primary antibodies. The protein signals were detected by chemiluminescence (Clarity Western ECL substrate, Bio-Rad, Milan, Italy); the densitometric analysis was obtained using the Molecular Imager ChemidocTM (Bio-Rad, Milan, Italy), and data were normalized against β-actin expression.

### 2.4. Determination of mtDNA Content

Total DNA was obtained from liver samples of rats belonging to the five experimental groups. MtDNA copy number relative to nuclear DNA was determined by quantitative real-time polymerase chain reaction (qPCR), as reported in Chimienti et al. [17]. The listed primers: mtDNA For 5′ GGTTCTTACTTCAGGGCCATCA 3′ (nt 15,785–15,806), mtDNA Rev 5′ TGATTAGACCCGTTACCATCGA 3′ (nt 15,868–15,847) (GenBankTM accession number AY172581); β-actin For 5′ CCCAGCCATGTACGTAGCCA 3′ (nt 2181–2200), β-actin Rev 5′ CGTCTCCGGAGTCCATCAC 3′ (nt 2266–2248) (GenBankTM accession number V01217.1) and 3 ng total DNA as template were used.

### 2.5. Modified Purines Analysis

Oxidized purines were detected using formamidopyrimidine DNA glycosylase (Fpg) (New England Biolabs, Beverly, MA, USA) digestion of total DNA, as in Chimienti et al. [18]. The PCR amplification of a 1000 bp-long amplicon encompassing the D-loop mtDNA region was conducted using the primers: For 5′ TCTGGTCTTGTAAACCAAAAATGA 3′ (nt 15,302–15,325) and Rev 5′ TGGAATTTTCTGAGGGTAGGC 3′ (nt 16,302–16,282) (GenBankTM accession number AY172581) and 5 ng of Fpg-treated or untreated total DNA as template. The ratio between Fpg-treated and untreated band intensities was evaluated, and results were expressed as the complement to 100%.

### 2.6. Statistical Analysis

GraphPad Prism software v8 was used for statistical tests. Due to the non-normal distribution of the data, nonparametric tests were performed. Statistical analyses were made using Kruskal-Wallis one-way analysis of variance with Dunn’s multiple comparisons post hoc tests. Differences were considered statistically significant at a value of *p* < 0.05. All data represent the results of at least two independent experiments and are expressed as mean ± SEM (standard error of mean).

## 3. Results

### 3.1. Diet-Related Mitochondrial Oxidative Stress

**Figure 1:**
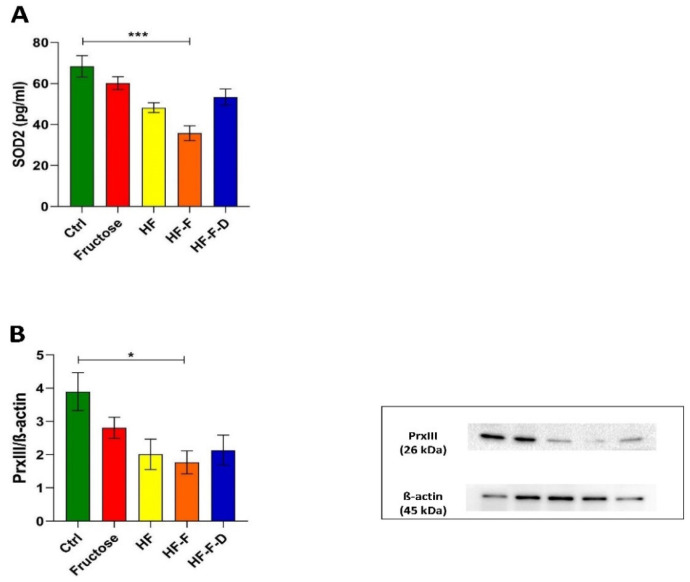
Superoxide dismutase (SOD2) concentration by Enzyme-Linked Immunosorbent Assay (ELISA) (**A**) and Western blot analysis of PrxIII amount (**B**) in liver samples from the Ctrl, Fructose, HF, HF-F, and HF-F-D rats. with each group consisting of four rats. Data were analyzed by Kruskal–Wallis analysis of variance and Dunn’s Multiple Comparison Test (* *p* < 0.05; *** *p* < 0.001), each group consisting of four rats.

The initial point was the detection of mitochondrial oxidative stress in the presently analyzed obesogenic diet model for NAFLD. Therefore, the concentration of the mitochondrial SOD2 and the amount of the ROS scavenger protein Peroxiredoxin III (PrxIII) were determined in the liver from rats belonging to the five experimental groups. All groups subjected to dietary interventions showed an evident decrease in both markers in comparison with the controls. In particular, the post-test revealed the significance of the difference only between the Ctrl and HF plus fructose diet (HF-F) groups (47% and 55% reduction in SOD2 and PrxIII, respectively). The fortification with DHEA increased the amounts of both mitochondrial oxidative markers compared to the HF-F counterpart, but without reaching statistical significance (Figure 1).

### 3.2. Analysis of MtDNA Relative Content and Oxidative Damage

**Figure 2:**
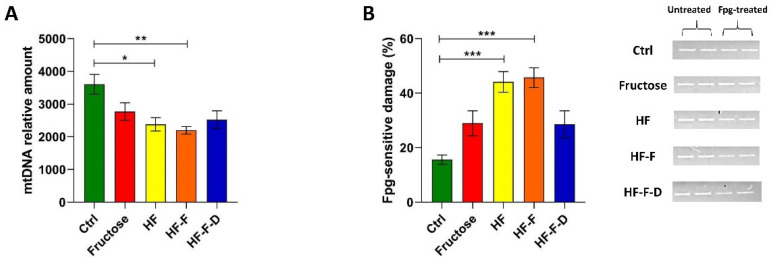
(**A**) MtDNA relative content in liver samples from Ctrl, Fructose, HF, HF-F, and HF-F-D rats. (**B**) Incidence of oxidatively modified purines at the D-loop in liver samples. Bars in the graph represent the ratio between Fpg-treated and untreated band intensities, expressed as the complement to 100%. A representative agarose gel showing amplicons obtained from Fpg-treated and untreated total DNA is reported in the right panel. (**A**,**B**) Data were analyzed by Kruskal–Wallis analysis of variance and Dunn’s Multiple Comparison Test (* *p* < 0.05; ** *p* < 0.01; *** *p* < 0.001), each group consisting of four rats.

Having verified the presence of the mitochondrial oxidative stress in all rat groups but the controls, the next step was the determination, by qPCR, of the relative content of mtDNA because this has been demonstrated to be affected in various ways by the organelle’s oxidative stress [16,17,19]. All dietary interventions induced a marked reduction in this parameter in comparison with the Ctrl counterpart. The difference reached statistical significance versus rats maintained on the HF and the HF-F diets (34% and 39% reduction, respectively). HF-F-D rats showed an increase in mtDNA relative content compared to the HF-F group, but this increase did not reach statistical significance (Figure 2A).

To find whether the dietary interventions also induced oxidative damage to mtDNA, total DNA was assayed for incidence of oxidized purines, mainly 8-oxo-deoxyguanosine (8-oxodG), through incubation with the oxidized purines-sensitive enzyme formamidopyrimidine DNA glycosylase (Fpg). The abundance of a 1000 bp-long amplicon, encompassing the D-loop region of mtDNA, was determined in Fpg-treated samples and their untreated paired samples. The incidence of 8-oxodG, the main product of the oxidation of purines, was significantly higher in HF and HF-F rats than in controls (2.8 and 2.9 times, respectively). Rats fed with the HF plus fructose diet fortified with DHEA showed an insignificant reduction of the incidence of oxidized purines compared with their HF-F counterparts (Figure 2B).

### 3.3. Evaluation of Mitochondrial Biogenesis Markers

To investigate whether mitochondrial biogenesis was affected by the dietary interventions, the amounts of peroxisome proliferator-activated receptor-γ co-activator-1α (PGC-1α), a master regulator of mitochondrial biogenesis, of mitochondrial transcription factor A (TFAM), the histonelike protein deeply involved in the maintenance of mtDNA, and of the COX-IV subunit from the cytochrome c oxidase complex, were evaluated. The COX-IV subunit is coded for by nuclear DNA, and its expression is usually coordinated with that of the mtDNA-encoded subunits of the same respiratory complex and directly regulated by PGC-1α.

**Figure 3:**
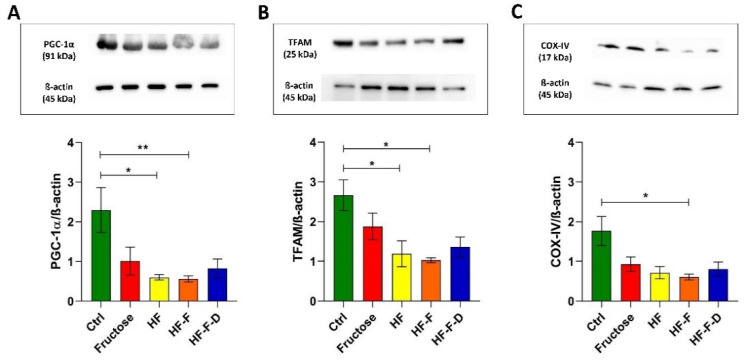
Western blot analysis of PGC-1α (**A**), TFAM (**B**), COX-IV (**C**) in liver samples from Ctrl, Fructose, HF, HF-F, and HF-F-D rats, with each group consisting of four rats. Data were analyzed by Kruskal–Wallis analysis of variance and Dunn’s Multiple Comparison Test (* *p* < 0.05; ** *p* < 0.01).

The amount of PGC-1α was affected by the dietary interventions, showing decreased values in rats subjected to dietary interventions with respect to those of Ctrl rats. The 74% and the 76% reduction found in HF and HF-F rats, respectively, were statistically significant. DHEA fortification showed a not significant increase in PGC-1α compared with the HF-F diet (Figure 3A).

All dietary interventions induced a decrease in TFAM amount statistically significant in HF and HF-F rats compared to Ctrl rats (55% and 61% reduction, respectively). The DHEA fortification induced a not significant increase compared to HF-F (Figure 3B).

As for the amount of COX-IV, the decreased values found in all diet groups achieved statistical significance only in the comparison between Ctrl rats and the HF-F group (66% reduction). Like the other analyzed markers, the amount of this protein, although increased in HF-F-D rats (33% increase compared to HF-F), was not significantly different from the HF-F counterpart (Figure 3C).

### 3.4. Analysis of Autophagy Markers

Having verified the negative effects of the tested dietary interventions on mtDNA maintenance and mitochondrial biogenesis, we analyzed the possible induction of autophagy as a cell response to the mitochondrial dysfunction by determining the amount of two proteins involved in this process. Therefore, the amounts of Beclin-1 involved in the early phase and the lipidated LC3 II form of the microtubule-associated protein 1 light chain 3 (LC3) were measured.

**Figure 4:**
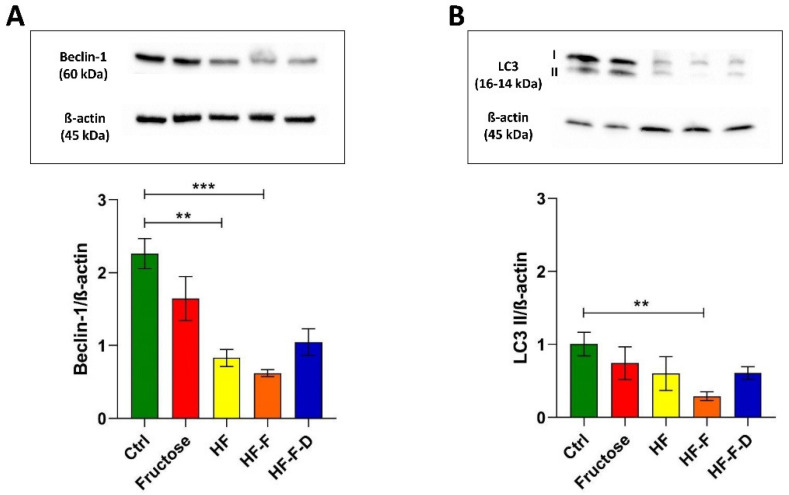
Western blot analysis of Beclin-1 (**A**), and LC3 (**B**) in liver samples from Ctrl, Fructose, HF, HF-F, and HF-F-D rats, with each group consisting of four rats. Data were analyzed by Kruskal–Wallis analysis of variance and Dunn’s Multiple Comparison Test (** *p* < 0.01; *** *p* < 0.001).

Both the investigated markers of autophagy were reduced in all rat diet groups compared with the values of Ctrl rats. Statistical significance of the differences was found for the Beclin-1 values between Ctrl and HF or HF-F (63% and 73% reduction, respectively) (Figure 4A) and the LC3 II values between Ctrl and HF-F (71% reduction) (Figure 4B). The fortification of the HF-F diet with DHEA increased the amounts of both markers of autophagy (Figure 4A,B, respectively) without reaching statistical significance.

## 4. Discussion

Due to the recent epidemiclike diffusion of NAFLD, several NAFLD/NASH animal models, mostly mice and rats, have been developed to help dissect the disease’s pathogenetic process [20,21,22]. Such models can be divided into diet-induced, genetic, or derived by combining different interventions [23]. In particular, since NAFLD’s prevalence has dramatically risen in concurrence with lifestyle change towards the adoption of an unhealthy Western-type diet, we decided to utilize a rat model mimicking obesogenic Westernized dietary habits through the combination of a high-fat diet with a high fructose intake (HF-F group) administered for 15 weeks [14]. Such a Western-type diet has also been shown to induce the activation of NLRP3 inflammasome that characterizes the inflammatory progression of NAFLD [24,25]. The high-fat diet (HF) components included both 26% fat and 4% cholesterol as a synergistic interaction between fatty acids and cholesterol derivatives has been shown to facilitate the onset and progression of NAFLD in diet-induced models [26]. The tested treatments also included a diet composed of 10% (*w*/*v*) fructose dissolved in the drinking water (Fructose group) because of the recent finding of increased fructose intake through the consumption of sugar-sweetened beverages and sugar-rich processed foods. Such a rise in the consumption of fructose has been associated with a higher prevalence of metabolic diseases, including NAFLD, because of the high lipogenic power of fructose, which is metabolized almost exclusively by the liver and strongly activates hepatic “de novo” lipogenesis [1]. In a previous study on the model of rats fed a high-fat diet with a high fructose intake, a subset of animals treated with the standard chow diet was also supplemented for 15 weeks with 0.01% (*w*/*w*) DHEA only, a multitargeted antioxidant compound tested against diet-induced oxidative damage [27], without showing any difference from the controls as for the assayed markers [14]. In another study by the same group [28] a DHEA dosage of 0.02% (*w*/*w*), which was twice that of our DHEA-supplemented group, was used in rats without implying any change in the tested parameters, also including oxidative stress markers. These considerations led us to use the DHEA dosage of 0.01% (*w*/*w*) directly in the HF-F group as a possible mean to counteract the diet-induced oxidative stress and its consequences. Effectively, the study focused on SREBP-1c involvement in the same rat model of NAFLD [14] showed that the dysregulation of triglycerides and free fatty acids (FFAs), leading to hepatic steatosis and induction of oxidative and inflammatory stress, favoring the progression of NAFLD, were markedly decreased in rats fed an HF-F, DHEA-fortified diet [14]. As for the specific antioxidant effect, DHEA administration was reported to decrease only H_2_O_2_ and HNE concentrations both in plasma and liver, and total SOD activity in liver [14] namely at cell and whole body levels.

However, the relevance of oxidative stress for the onset and progression of NAFLD has been extensively demonstrated in recent reviews [8,23,29], and mitochondria are the major, although not unique, generators of reactive oxygen species (ROS) in cells. Excess ROS are produced in hepatic mitochondria of animals exposed to an obesogenic diet by two concurrent processes: the increased β-oxidation of FFA and the downregulation of the electron transport chain (ETC), which stimulates ROS overproduction upstream of cytochrome c oxidase. The first process is due to the excessive delivery of FFA, deriving from the uninhibited lipolysis in adipose tissue. The lipolysis of triglycerides in adipocytes is regulated by insulin, but a low insulin sensitivity in peripheral tissues characterizes the IR, a feature of NAFLD pathogenesis. Other extra FFAs derive from hepatic “de novo” lipogenesis, through which the surplus dietary energy, mostly introduced as carbohydrates and fat, is partially used, leading to the massive deposition of triglycerides and resulting steatosis [30]. The imbalance of ETC activity in NAFLD models and patients has been largely described in the literature and thoroughly examined [31]. In the latter study, the activities of ETC complexes were analyzed and found to decrease for all complexes but Complex II in the NAFLD model. This result was explained through the lipid-rich condition, fueling electrons from β-oxidation to ETC by mitochondrial complex II. The same study also assessed oxidative stress at the mitochondrial level by determining the amounts of lipoperoxidation markers and the total quantity of oxidized proteins inside the organelles. Due to the considerable relevance of mitochondrial oxidative stress for the onset and progression of NAFLD, we decided to evaluate whether crucial functions as maintenance of mtDNA, mitochondrial biogenesis, and autophagy were affected in the obesogenic diet-fed rat model of NAFLD.

The starting point was the confirmation of the diet-induced oxidative stress at mitochondrial level, in our model, through analysis of the amounts of the mitochondrion-specific SOD2 and H_2_O_2_-scavenging enzyme, PrxIII. In the present study, all tested diets led to a decrease in SOD2 and PrxIII amounts, which was significant in the HF-F group and was not prevented in the HF-F-D rats, thus demonstrating the mitochondrial oxidative stress induced by all diets. These findings agree with other reports showing marked oxidative stress inside mitochondria because of the obesogenic diets administered [23,31]. In particular, in liver from genetically modified mice, placed on an obesogenic diet, the diminished expression and activity of SOD2 have been described [32].The reduction of the mitochondrial scavenger protein PrxIII has been reported in various conditions featuring oxidative stress inside the organelles [33]. Previous studies have demonstrated that depletion of PrxIII resulted in increased intracellular levels of H_2_O_2_ and sensitized cells to induction of mitochondria-mediated apoptosis [34]. Furthermore, some of us reported an age-related decrease in PrxIII expression in rat liver prevented by a caloric restriction diet [32]. The reduced amounts of SOD2 and PrxIII, found in all rat groups, can be explained by the hypothesis that the diet-induced oxidative stress was so serious/long-lasting as to lead to the exhaustion of the initial compensatory response of the mitochondrial antioxidant system as suggested in other reports [30]. Effectively, recent studies, performed in cell culture, animal models or human patients, show mitochondrial ROS production and its causal role in the oxidative damage of NAFLD [34]. In particular, a pro-oxidative condition appears to precede extensive mitochondrial damage and the subsequent mitochondrial impairment in NAFLD pathology [35]. In this same study a very appealing hypothesis about the sequential events leading to mitochondrial dysfunction and a facilitated onset of NAFLD in patients was presented. In the liver of obese humans, with and without steatosis, the increased lipid availability should stimulate hepatic mitochondrial capacity to prevent NAFLD progression. However, mitochondria have been demonstrated to leak and their increased respiration should not be bioenergetically efficient, but rather promote excessive oxidative stress, challenging antioxidant defense mechanisms. Once these mechanisms have failed, mitochondrial functionality should reduce and promote liver insulin resistance, NAFLD progression to NASH, and systemic inflammation. The author suggested the existence of a ‘‘hepatic metabolic flexibility’’ mechanism by which liver mitochondria should adapt to altered bioenergetic demands at an early phase of the impaired lipid metabolism. Later on such flexibility should be lost, because of the overwhelming lipid-induced oxidative stress, and promote the other cell changes leading to the progression from NAFLD to NASH [36].

The failure of the mitochondrial antioxidant response and the overwhelming oxidative stress may open the way to a serious dysfunction of the organelles that might impinge on other vital functions such as the maintenance of mtDNA and biogenesis. Therefore, we checked the relative content of mtDNA as this marker had been shown to suffer from various oxidative stress-associated conditions [16,17,19]. All diets induced a decrease in mtDNA content that was statistically significant in the HF and HF-F groups. Fortification with DHEA did not prevent such a decrease, as shown in a previous study on mitochondrial mass in this NAFLD animal model [37]. In agreement with our results, the mtDNA content was shown to be decreased in obese NAFLD and NASH patients in comparison to controls and obese without steatosis [36] and in another study comparing mtDNA from NAFLD/NASH patients and controls [38]. In particular, the latter paper showed the accumulation of mutations in the mtDNA of patients and the association between disease severity assessed at histological level and the total number of mutations in OXPHOS genes [38], thus further supporting the possibility that the original mitochondrial oxidative stress might be reinforced by the dysfunction of the mutated mtDNA-encoded subunits of ETC. The mtDNA content has been reported, according to the mitohormesis theory, to be sensitive to mitochondrial oxidative stress in a biphasic way, namely, mild oxidative stress may induce an increase in mtDNA content, and vice versa, severe oxidative stress may lead to the loss of mtDNA [16]. The size of the diet-induced decrease in the mtDNA content, shown here, strongly suggests serious mitochondrial oxidative stress at the beginning of NAFLD. The seriousness of the diet-induced mitochondrial oxidative stress was further verified by determining the incidence of 8-oxodG in the D-loop region of mtDNA, which was significantly higher in HF and HF-F rats than in controls (2.8 and 2.9 times, respectively). In the HF-F-D group DHEA did not prevent the oxidative damage of mtDNA. It has been recently demonstrated that oxidized mtDNA can lead to the activation of NLRP3 inflammasome [39] and to the inflammatory progression of the disease. To give a rough idea of the meaning of these values, it can be noted that the age-related increase in the incidence of 8-oxodG in the same D-loop region of mtDNA from standard chow-fed rat liver, passing from 18 months to 28 months of age, was only about 10% [17]. With such serious oxidative damage to mtDNA, it was necessary to evaluate mitochondrial biogenesis by determining two proteins fundamental for the amplification process leading to regulated expression of mtDNA-encoded proteins, namely PGC-1α, the master regulator of mitochondrial biogenesis, and TFAM, the histonelike protein of mtDNA. There is significant evidence about the redox sensor role of PGC-1α, and mitochondrial ROS may control PGC-1α expression, thus linking it tightly to NAFLD pathogenesis [40]. All dietary interventions induced a marked decrease in PGC-1α, which reached statistical significance in the HF and HF-F groups, respectively. The DHEA fortification did not prevent the diet-induced decrease in the protein and likely in mitochondrial biogenesis. The negative effect of all diets on mitochondrial biogenesis was further confirmed by the statistically significant decrease in TFAM amount in HF and HF-F rats. Again, the DHEA fortification did not prevent the diet-induced decrease. The decreased values found for the amount of COX-IV in all diet groups confirmed that the reduction of mitochondrial biogenesis affected the nuclear DNA-encoded mitochondrial proteins, which are generally expressed in a coordinated way with the mtDNA-encoded proteins to allow the physiological assembling of the mitochondrial ETC complexes. Again, in this instance, the DHEA supplementation did not prevent the diet-induced decrease in COX-IV amount. PGC-1α also coordinates the autophagic removal of damaged components [41], and autophagy is generally stimulated by oxidative stress [1]. Therefore, we assayed autophagy by determining the amounts of Beclin-1, a protein at the beginning of the autophagic pathway, and the phosphatidylethanolamine-bound form (LC3 II) of LC3, which correlates to autophagosome levels. The diet-induced reduced amounts of Beclin-1 and LC3 II support the loss of efficient autophagy in all these animals. DHEA supplementation was not able to promote the autophagic elimination of damaged components. This finding further suggests that the diet-induced mitochondrial oxidative stress was so serious as to lead to a dramatic dysfunction of the organelles that could not be fixed by deranged autophagy [1]. Consistently, reduced autophagy has been reported in fatty liver and hepatocytes’ autophagy has been shown to provide resistence to liver injury in NAFLD and protect from progression to NASH in diet-induced models. Therefore, the derangement of autophagy appears to be quite relevant for NAFLD pathophysiology [42] together with oxidative stress. In particular, a relevant mitochondrial oxidative stress can induce enzyme inactivation by protein oxidation, alterations and depletion of mtDNA. Furthermore, oxidative damage to nuclear DNA may amplify mitochondrial impairment by compromising the transcription of critical mitochondrial proteins as PGC-1α, NRF-1 and TFAM. ROS can also “attack” polyunsaturated fatty acids, leading to the production of aldehyde byproducts, namely, MDA and HNE, that can diffuse from their origin, amplifying the effects of oxidative stress [35]. Two more mechanisms able to enhance the hepatic damage related to oxidative stress are the gut–liver axis and endothelial dysfunction. As for the gut–liver axis, some recent studies have verified intestinal dysbiosis as a factor leading to increased liver lipotoxicity in animal models of NAFLD [29]. Additionally, the described imbalance in eNOS and iNOS functionality drives an increase in oxidative damage of the liver vasculature leading to endothelial dysfunction, inflammation and fibrosis [29].

The present results demonstrate the relevance of mitochondrial oxidative stress and the sequential dysfunction of the organelles in our obesogenic diet-induced animal model of NAFLD, thus fostering the strong relevance of oxidative stress for the onset and worsening of the disease.

The absence of positive results from the DHEA treatment can be explained if the DHEA did not reach mitochondria where the oxidative stress had already blown up with dramatic consequences for the organelle metabolism. We suggest that DHEA can delay the progression from NAFLD to NASH by buffering, through its antioxidant effects, the amplification at cell level of the oxidative stress, acting on those markers related to processes downstream of the mitochondrial oxidative stress, such as the inflammatory process and fibrosis. Clarifying mitochondrial processes might be crucial for the resolution of NAFLD and its progression to NASH [43,44], especially because at present, NAFLD is tentatively counteracted through the adoption of measures requiring a patients’ compliance that is reached and maintained with great difficulty, namely diet and an active lifestyle [45]. Future studies are also required to identify pharmaceuticals that might target mitochondrial dysfunction in NAFLD and thus act in the initial phases of the pathogenetic process.

## 5. Conclusions

Present results assessed the relevance of mitochondrial oxidative stress and the sequential dysfunction of the organelles in our HF-F-fed animal model of NAFLD. The marked, diet-induced mitochondrial oxidative stress was demonstrated by the decrease in SOD2 and PrxIII amounts, the decrease in mtDNA content and the increase in its oxidative damage. The diet’s negative effect on mitochondrial biogenesis was shown by decreased amounts of PGC-1α, TFAM, and COX-IV subunit. The reduced amounts of Beclin-1 and LC3 II demonstrated the diet-related autophagy’s decrease. The DHEA supplementation did not prevent the diet-induced changes probably because the DHEA did not reach mitochondria where the oxidative stress had already blown up with dramatic consequences for the organelle metabolism.
